# À propos d’un cas d’oreilles décollées

**DOI:** 10.11604/pamj.2019.34.161.19995

**Published:** 2019-11-26

**Authors:** Karim Bourra, Nabil Berny

**Affiliations:** 1 University Hassan II Hospital Ibn Rochd, Casablanca, Morocco

**Keywords:** Otoplastie, oreilles décollées, esthétique de l'oreille, Otoplasty, sticking-out ears, ear aesthetic

## Abstract

We report the case of a 35-year old patient with sticking-out ears who had no particular past medical history. The patient had congenital ear anomaly which had progressed over the past years and that was a heavy psychological burden with serious social repercussions as well as a family and professional impact. Approach was based on surgery in order to provide the best possible outcomes. Ear surgery, also known as otoplasty, gives patients a natural appearance. In our case study, surgical outcome was satisfactory. The duration of surgery was 2 hours. The surgical procedure was based on ear-lobe repositioning with posterior lift of the skin and reshaping of the conch in order to fix it back to the bone. The patients needed dressing change every other day. He had the stitches removed on day 10.

## Image en médecine

Patient de 35 ans sans antécédents pathologiques particuliers, dont l'oreille est décollée et présente une anomalie à l'oreille qui évolue depuis la naissance et qui représente un lourd fardeau psychologique avec de graves répercussions sociales avec impact familial et professionnel. La marche à suivre consistait à opérer le patient en obtenant un état acceptable de ses oreilles. L'opération appelée otoplastie consiste à donner aux oreilles un front aussi proche de l'état normal. Nous présentons notre cas opéré avec un résultat satisfaisant. La durée de l'opération était de 2 heures. Repositionnez le lobe de l'oreille avec un lifting postérieur de la peau et tirez sur conque pour la fixer en arrière sur l'os. Le gros pansement a changé 1 jour sur 2. Ablation du fil à j10. Résultat opérationnel à j10 (face+profil).

**Figure 1:**
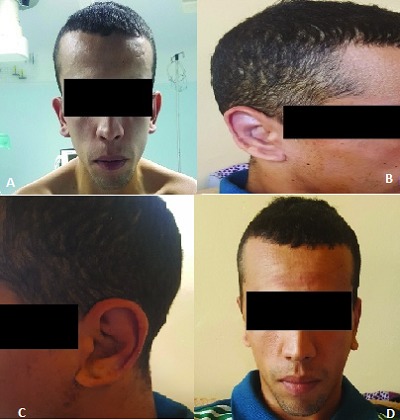
A) avant opération vue de face; B) après opération oreille gauche de profil; C) après opération oreille droite de profil; D) après opération a j10 vue de face

